# SNP2Structure: A Public and Versatile Resource for Mapping and Three-Dimensional Modeling of Missense SNPs on Human Protein Structures

**DOI:** 10.1016/j.csbj.2015.09.002

**Published:** 2015-09-30

**Authors:** Difei Wang, Lei Song, Varun Singh, Shruti Rao, Lin An, Subha Madhavan

**Affiliations:** a Department of Oncology, Lombardi Comprehensive Cancer Center, Georgetown University Medical Center, Washington, DC 20007, USA; b Innovation Center for Biomedical Informatics, Georgetown University Medical Center, Washington, DC 20007, USA; c Department of Biochemistry and Molecular & Cellular Biology, Georgetown University, Washington, DC 20007, USA

**Keywords:** Active site mutations, Protein structure, Molecular modeling, Disease causing SNPs, SNP database

## Abstract

One of the long-standing challenges in biology is to understand how non-synonymous single nucleotide polymorphisms (nsSNPs) change protein structure and further affect their function. While it is impractical to solve all the mutated protein structures experimentally, it is quite feasible to model the mutated structures in silico. Toward this goal, we built a publicly available structure database resource (SNP2Structure, https://apps.icbi.georgetown.edu/snp2structure) focusing on missense mutations, msSNP. Compared with web portals with similar aims, SNP2Structure has the following major advantages. First, our portal offers direct comparison of two related 3D structures. Second, the protein models include all interacting molecules in the original PDB structures, so users are able to determine regions of potential interaction changes when a protein mutation occurs. Third, the mutated structures are available to download locally for further structural and functional analysis. Fourth, we used Jsmol package to display the protein structure that has no system compatibility issue. SNP2Structure provides reliable, high quality mapping of nsSNPs to 3D protein structures enabling researchers to explore the likely functional impact of human disease-causing mutations.

## 1 Introduction

Next-generation sequencing (NGS) has enabled the rapid discovery of single nucleotide polymorphisms (SNPs) in humans [1]. SNPs account for about 90% of human genetic variation. Most of this genetic variation does not affect protein structure and function. However, non-synonymous SNPs (nsSNPs), which change the amino acid sequence of a protein, usually have a detrimental effect on protein structure and/or function and are frequently associated with human diseases.

In the last decade, considerable effort has been devoted to using nsSNP information to mapping the mutations onto the protein sequence and to predict potential functional impact and the association with human diseases [2], [3], [4], [5], [6], [7], [8], [9], [10], [11], [12], [13]. Although it is possible to predict disease causing effects for nsSNP changes with about 70 to 90% accuracy using various annotated databases (2, 5 –7, 11a), it is still challenging to understand such deleterious effects at the protein structure level. Several web portals have been built to predict the effects of mutations on protein function and association with disease [7], [11], [13]. These resources are limited in their visualization capabilities and accessibility; there is a need to build a more user-friendly resource to provide direct mapping of nsSNPs onto 3D protein structural models. Protein structure visualization provides critical visual information about how mutations impact biological protein function and helps to guide drug design and discovery.

The challenge of understanding how nsSNPs are associated with disease motivated us to build a database for all known human protein structures with modeled nsSNP mutation sites as a freely available resource for the scientific community. As our first step toward understanding the deleterious effects of protein mutations, we developed a novel pipeline to map all nsSNPs mutations in dbSNP (build 137) [14] onto the available human X-ray and solution NMR structures in the Protein Data Bank (PDB) [15]. In particular, we are focusing on missense SNPs (msSNP, a common type of nsSNPs). Our mapping pipeline addressed shortcomings in some of the existing web portals with a similar aim. We found that some of the existing web portals do not provide three-dimensional structures but instead are using static images of the structures [13]. The ability to visualize residue mutations in 3D structures enables users to see if the mutation alters catalytic activity due to its proximity to the active site; to determine if the mutation alters key interactions inside the protein itself or between macromolecules; or if the mutation changes the stability of the folded structure. We found inconsistent residue numbering between PDB structures [15] and UniProt sequences [16], which can result in a position shift in mapping the mutated residue to the wild type structure [13]. The mapping discrepancies within these two resources have resulted in errors being propagated into derivative databases and could potentially lead to incorrect assignment of mutation site in the structure of interest based on these databases. It is not a trivial task to correct the discrepancies [17].

A significant novel feature in SNP2Structure is the direct comparison of two protein structures (wild type vs. mutated or mutated vs. mutated) with a user-friendly interface. SNP2Structure also provides information on all associated and interacting molecules (protein, DNA, RNA and small ligands) packed in the original crystal structure for displaying protein models, in contrast to other resources that focus on the protein structure only [7], [11], [13]. Our tool allows users to inspect protein mutations that may affect the interaction with other macromolecules. The implantation of Jsmol package (http://chemapps.stolaf.edu/jmol/jsmol/jsmol.htm) avoids the system compatibility issue for displaying protein structure. Finally, all mutated structures are downloadable for further structural analysis. We believe SNP2Structure will be a valuable resource for exploring potential structural and functional impact of missense mutations in various human diseases.

## 2 Methods

**Fig. 1:**
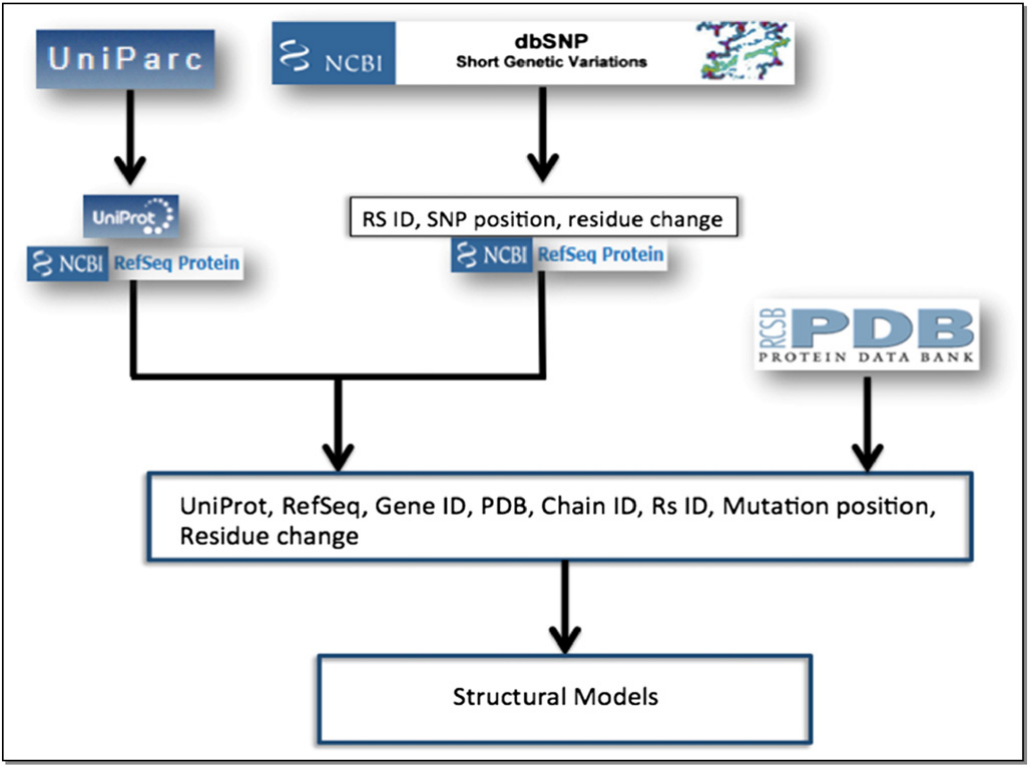
Scheme of structural model building using publicly available resources.

SNP2Structure is based on the integration of data from publically available bioinformatics resources to annotate mutated positions both in protein sequences and their associated X-ray and solution NMR structures. We took advantage of the existing mutation information in dbSNP and the annotation of protein sequences in UniProt and UniParc [16], (Fig. 1) to obtain the genomic location of each msSNP.

To obtain protein sequence information, we parsed the UniParc XML file to retrieve the human sequences and their UniProt and RefSeq IDs with active status [18]. No isoforms were considered. We then parsed the dbSNP gene records to get the RefSeq IDs and the corresponding dbSNP Reference SNP IDs (rs IDs), mutation positions, and residue name. Only mutations having a single amino acid change were considered. Indels, frame-shift, and synonymous mutations were discarded. These two lists were merged together and only the matched RefSeq IDs were stored for further analysis. The residue positional information in our dataset refers to the ‘canonical’ sequence in UniProt. The reasons we selected UniProt as our reference database are: 1) UniProt is a highly curated and manually reviewed database, and 2) UniProt has a strict rule to select the canonical sequence for all the protein products encoded by one gene. To obtain protein structure information, we retrieved all existing human protein structures in PDB and only considered the X-ray and solution NMR structures. We then merged these two lists (sequence and structure) by filtering out unmatched UniProt IDs.

Based on our findings, approximately 5 –10% of protein structures in PDB have incorrect amino acid residue numbering compared to the annotations in UniProt. This was a critical problem to address since the discrepancies can lead to errors when mapping mutation sites to protein structures. For example, the sequence of Alcohol Dehydrogenase 5 (ADH5) in the UniProt entry (P11766) contains residues Gly60, Cys61 and Phe62. However, in one of the ADH5 X-ray structures (PDB 1TEH), it has Gly60 followed by Cys60A and Phe61. The unconventional residue number Cys60A in PDB 1TEH is the cause of the residue numbers after Gly60 being changed by − 1, which is a problem if a single amino acid mutation occurs at any residues after Gly60. Two other examples include structures of Alcohol Dehydrogenase class 4 mu/sigma chain (ADH7), PDB 1AGN and 1D1S1AGN1D1S. In these structures, Thr118 directly follows Ile116. It looks like residue 117 is missing in the structures. When we aligned the actual PDB sequences to the corresponding UniProt sequence (P40394), we found that they correspond to Ile129 and Thr130, respectively. There is no missing residue between Ile129 and Thr130. It is clear that the difference between PDB numbering and UniProt numbering is not consistent from the start to the end residue. These discrepancies cause errors when assigning the mutation positions in the structures. For instance, rs59534319 corresponds to the Lys238Glu mutation of the ADH7 protein in UniProt. In one web portal instead of Lys226 (there is a 12 difference between UniProt and PDB residue numbering), it was assigned to residue 225 in PDB 1D1S due to the inconsistencies between UniProt and PDB [13].

To correct the problem of residue numbering in PDB structures, we aligned the actual PDB sequences to the corresponding UniProt sequences and reassigned the residue numbers in PDB structures according to residue numbers in UniProt sequences. The actual PDB sequences were generated by parsing the ATOM records in the PDB files. The needle program in the EMBOSS package [19] was used for sequence alignment between actual PDB sequence and the corresponding UniProt sequence. Only the structures without mutations and/or missing residues compared to its UniProt canonical sequence and more than 20 amino acids long in length in the mapped range were considered in the current release of SNP2Structure. Next, we renumbered all considered PDB structures with the correct residue numbers based on sequence alignment results. We then used the final table with all the information as input to build the structural models using Modeller 9.11 [20] (~ 280,000 models). The model building procedure is straightforward. Only the coordinates of atoms in the side chain of the mutated residues were changed. The rest of the coordinates were kept intact. We have built one model per mutation per PDB entry. For evaluating the mutation effect of msSNPs, we either calculated the PolyPhen2 score or parsed the SIFT results table. PolyPhen2 and SIFT are the two popular tools to predict potential impact of an amino acid substitution on the protein structure and function of a human protein [5], [6]. PolyPhen2 uses straightforward physical and comparative considerations while SIFT calculates the probability of the amino acid change at certain position in a queried sequence after comparing it with related protein sequences. These scores indicate if the mutation is deleterious or not. SNP2Structure also includes DrugBank ID for small molecules, active site, and metal binding site information. The active site/metal binding site information was extracted from the UniProt database. For comparison, we also included resolution for X-ray structure, chain information, and other key information in the database. In addition, the relative solvent accessible area for both wild-type and mutated structures were calculated using the NACCESS 2.1.1 program [21].

## 3 Results

### 3.1 Data Portal Features

**Fig. 2:**
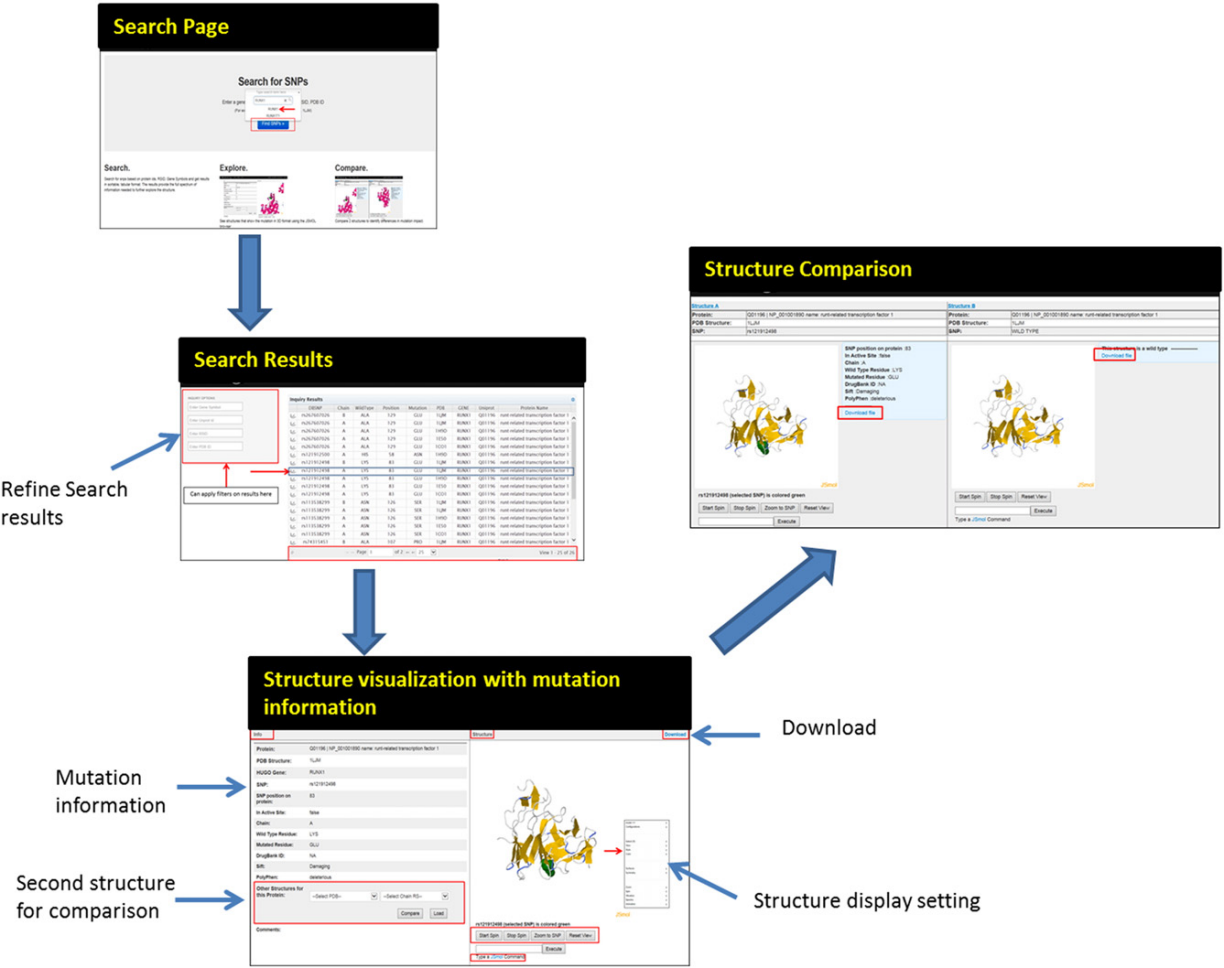
Key components of SNP2Structure database.

SNP2Structure was designed to be a user-friendly and web-based portal. It has four key components: 1) search, 2) structural visualization, 3) structural comparison, and 4) download. The protein mutation information can be queried through an Oracle database (Fig. 2). The corresponding structures/models can be downloaded in PDB format for further analysis.

### 3.2 Search

Users are able to retrieve protein mutation information of interest by inputting the HUGO gene symbol, UniProt ID, dbSNP rs ID, or PDB ID in the search box on the homepage. For example, enter HUGO gene symbol RUNX1 [Runt-related transcription factor 1, also known as acute myeloid leukemia 1 protein (AML1) or core-binding factor subunit alpha-2 (CBFA2)] in the Search box. If it exists in the database, any record that contains RUNX1 as a string/sub-string will appear while the user is typing. Select the correct gene and click the button with “Find SNPs.” For RUNX1, it outputs 26 msSNP mutation records. The output table is downloadable as a text file. If your query does not exist in the database, it will report a page with a “no records existing” message.

### 3.3 Structural Visualization and Comparison

The structural visualization component allows users to visualize protein structures of interest. The default display setting for protein is ribbons colored by secondary structures. Unlike existing portals, our portal provides an interactive display of the 3D protein structures in JSmol. By simply right clicking on the structure, a user can access various features in JSmol, such as, changing the style and color of the structure, highlighting hydrogen molecules and water molecules, highlighting the van der Waals surface on the protein structure, zoom in and out of a mutation site, spin the structure, etc. A user can also utilize the command line box to execute these functions. Moreover, hovering over the secondary protein structures will display the amino acid residue name and number.

Since we used the structure in the asymmetric unit to generate the models, users may explore the likely interactions between mutated proteins and their associated macromolecules and ligands. Interactions, however, may be artificial due to the well-known crystal packaging effect. The use of biological assembly structures would be ideal, but they need extensive manual curation since more than one biological assembly model (author suggested, software calculated or both) may exist for a particular protein. The predicted results of PolyPhen2 and SIFT for the effect of mutations are displayed on the same page for reference. We also provide the relative surface area of the structures, which allows users to explore 1) the effect of a mutated structure compared to the wild type and 2) compare the effect of two different mutations on same structure.

In order to compare the structures of different mutations within the same protein, we designed two visualization windows. Side-by-side comparison can be done for either wild type structure vs. mutated structure or mutated vs. mutated structures for the same protein. For proteins with more than one X-ray structure, it is also possible to compare the mutated structures among those X-ray structures. This feature is valuable since it shows which parts of the protein structures may be conformationally variable.

### 3.4 Download Option and Portal Statistics

Users can download structures to their local computer or do analysis online. The search results can be exported as a table with all necessary information for the corresponding model retrieval. For example, the model with rs183443805 from PDB 1PML can be downloaded using the following link, https://apps.icbi.georgetown.edu/molecule3D/snp2str/DEC18/models/P00750_1PML_A_183443805_ARG_224_CYS.pdb. Since we have corrected the discrepancies between residue numbering between PDB and UniProt, the downloaded structures will have the corrected residue numbering. If users need the structures with original numbering, they shall visit the PDB website (http://www.rcsb.org) to download the original PDB files.

Our portal includes 1810 unique UniProt IDs, 7021 wild type protein structures (6135 X-ray and 886 NMR), and 26,097 unique dbSNP rs IDs. The total number of models is 289,709.

### 3.5 System Implementation

**Fig. 3:**
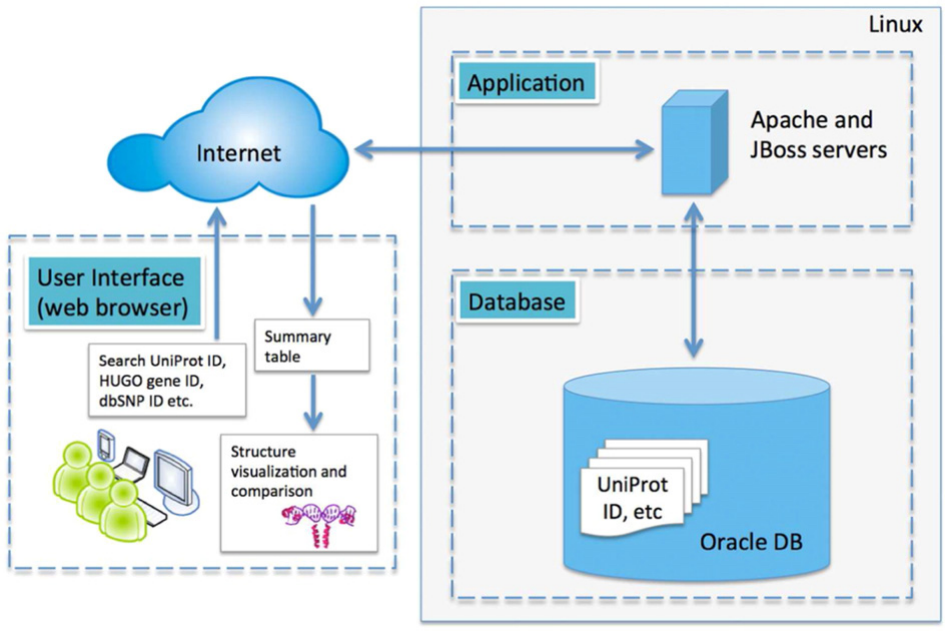
SNP2Structure web portal architecture.

The SNP2Structure database was developed under the Linux system using common software packages for the web server including Apache web server and Oracle database management, Fig. 3. The web interface of SNP2Structure was written in Groovy using the Grails framework. Data is stored in an Oracle database. We also used the JSmol package that is a JavaScript version of Jmol for protein structure visualization (http://chemapps.stolaf.edu/jmol/jsmol/jsmol.htm). The advantage of using JSmol instead of Jmol is that any HTML5 compatible web browser can open the JSmol application. Jmol also needs the Java Virtual Machine (JVM) installed first and often causes compatibility issues [7], [9]. We designed the system as simple as possible and made the database flexible, interactive, and intuitive for users. We recommend using Google Chrome, Firefox, or Safari to open the portal. The portal is not compatible well with Internet Explorer on a PC running Windows XP or Windows 7. SNP2Structure runs on m1.large instance (4 cores of CPUs with 7.5 Gb memory) on the Amazon cloud.

### 3.6 Application Example

**Fig. 4:**
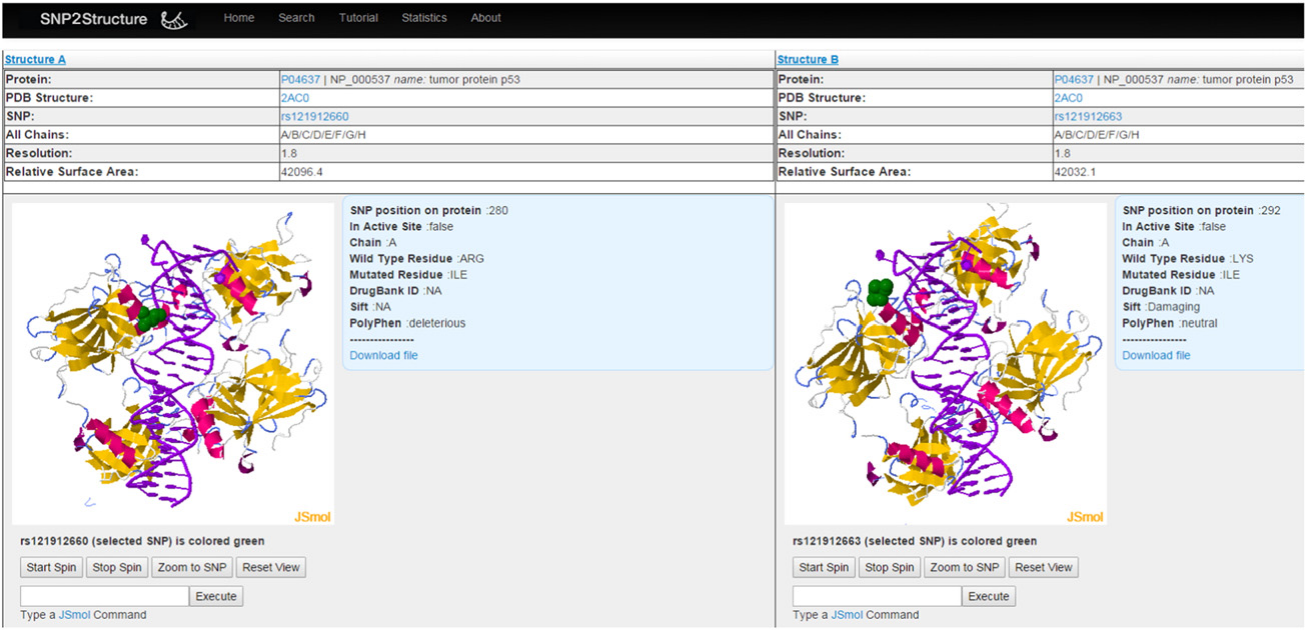
Comparison of two single amino acid mutations of p53 (Left: Arg280Ile and Right: Lys292Ile). DNA is shown as purple strands while p53 is displayed as colored ribbons. The mutated residues are displayed as green balls.

Here, we give an example to illustrate structural comparison, one of the unique applications of SNP2Structure. P53 is the most studied tumor suppressor protein in cancer biology. Hundreds of missense mutations in p53 have been identified in the last two decades. One of the most frequently mutated residues is Arg280, which makes hydrogen-bond interactions with Gua in the p53 canonical DNA response element CATG. Arg280Ile (rs121912660) (green balls, left, Fig. 4) mutation abolishes such important interactions and has deleterious effects on p53 tumor suppression function. Another less frequent p53 mutation Lys292Ile (rs121912663) seems to have a less deleterious effect since Lys292 (green balls, right, Fig. 4) is far from the DNA major groove. The PolyPhen prediction for Arg280Ile is deleterious and for Lys292Ile is ‘neutral’. Moreover, the relative surface area for the protein structure with mutation at Arg280Ile is 42096.4 compared to 42032.1 for the structure with a mutation at Lys292Ile.

## 4 Conclusion

We have built a web portal to share, visualize, and analyze protein structures constructed in-house that are associated with msSNP mutations. Our web application features direct comparison of two related structures: either wild type vs. mutated, or mutated vs. mutated. In addition, we carefully corrected the numbering discrepancy of mutation positions between structures and sequences using public resources. It is user-friendly and the structural models are downloadable for further structural and functional analysis. We believe this resource is valuable to the basic research community for understanding and exploring the likely functional impact of human disease-causing msSNPs as well as to translational researchers exploring structure-based drug design.

## Funding

This work was supported by the Food and Drug Administration (FDA) Centers of Excellence in Regulatory Science and Innovation (CERSI) program: [grant number FDA U01FD00413]; and the National Institutes of Health/National Cancer Institute [grant number U54-CA149147]. We also thank Amazon Corporation for providing the computing resources for this study.

## Conflict of Interest

None declared.
